# PSYCHE—A Valuable Experiment in Plant NMR-Metabolomics

**DOI:** 10.3390/molecules25215125

**Published:** 2020-11-04

**Authors:** Pauline Stark, Caroline Zab, Andrea Porzel, Katrin Franke, Paride Rizzo, Ludger A. Wessjohann

**Affiliations:** 1 Department of Bioorganic Chemistry, Leibniz Institute of Plant Biochemistry, Weinberg 3, 06120 Halle/Saale, Germany; carolinezab.95@gmail.com (C.Z.); Andrea.Porzel@ipb-halle.de (A.P.); Katrin.Franke@ipb-halle.de (K.F.); 2 Department of Molecular Genetics, Leibniz Institute of Plant Genetics and Crop Plant Research, Corrensstraße 3, 06466 Seeland, Germany; rizzo@ipk-gatersleben.de

**Keywords:** Pure Shift NMR, PSYCHE, *Hypericum*, metabolomics

## Abstract

^1^H-NMR is a very reproducible spectroscopic method and, therefore, a powerful tool for the metabolomic analysis of biological samples. However, due to the high complexity of natural samples, such as plant extracts, the evaluation of spectra is difficult because of signal overlap. The new NMR “Pure Shift” methods improve spectral resolution by suppressing homonuclear coupling and turning multiplets into singlets. The PSYCHE (Pure Shift yielded by Chirp excitation) and the Zangger–Sterk pulse sequence were tested. The parameters of the more suitable PSYCHE experiment were optimized, and the extracts of 21 *Hypericum* species were measured. Different evaluation criteria were used to compare the suitability of the PSYCHE experiment with conventional ^1^H-NMR. The relationship between the integral of a signal and the related bin value established by linear regression demonstrates an equal representation of the integrals in binned PSYCHE spectra compared to conventional ^1^H-NMR. Using multivariate data analysis based on both techniques reveals comparable results. The obtained data demonstrate that Pure Shift spectra can support the evaluation of conventional ^1^H-NMR experiments.

## 1. Introduction

Metabolomics is understood as a quantitative and comprehensive analysis of metabolites in a complex biological specimen to describe the chemical phenotype [1]. Nuclear magnetic resonance (NMR) spectroscopy and mass spectrometry (MS) are the key technologies used for that purpose. NMR is particularly convincing due to its simple sample preparation process, high reproducibility, and non-destructive character [2,3]. Originally, it was the method of choice for the structure elucidation of pure compounds; however, NMR is increasingly used nowadays for the analysis of complex mixtures [4]. In particular, the ability to quantify different metabolites at various concentration levels makes ^1^H-NMR a valuable tool for metabolite profiling and the fingerprinting of biofluids [5], foods [6] and natural product sources such as plants [7,8], fungi [9] and corals [10,11]. 

Plant metabolomics is challenging because, in addition to the primary metabolites, each species includes a high number of secondary metabolites, which helps the organism to interact with its environment. It is assumed that the metabolome of the plant kingdom comprises over 200,000 metabolites [1]. The genus *Hypericum* includes around 450 chemically diverse species [12]. The best-known species is *H. perforatum* (St. John’s Wort), which is commercially used against mild to moderate depression in the Western World [12,13]. In general, *Hypericum* species are characterized by several secondary metabolite classes, such as naphthodianthrones, phloroglucinols, flavonoids and xanthones [7,14,15,16]. In the ^1^H-NMR, those secondary metabolites and primary metabolites can be detected simultaneously [7]. However, the high number of signals from complex mixtures in the ^1^H-NMR spectrum is limited to a small spectral range, which leads to overlapping signals. It is further strengthened by proton–proton scalar couplings, which cause multiplet structures. Regions with overlapping signals complicate spectral analysis and identification [17,18,19]. 2D-NMR methods enhance the spectral resolution by spreading the overlapped signals in a second dimension, which is useful in structure elucidation. However, due to the long acquisition times required for most of the experiments, they were rarely applied in metabolomics investigations [2,20,21]. Homonuclear broadband decoupling methods, also called “Pure Shift” methods, were stated by Aquilar et al. as a possible “resolution of the resolution problem” [22]. They offer enhanced resolution by removing the effects of proton–proton scalar couplings and turning multiplets into singlets [23,24]. During the last few decades, different methods have evolved, such as 2D *J*-resolved NMR [25], slice selective decoupling (Zangger–Sterk) [19] and PSYCHE (Pure Shift yielded by chirp excitation) decoupling [26,27].

Besides all the theoretical benefits that Pure Shift methods promise for metabolomics experiments, a major bottleneck is still the low sensitivity, which (depending on the method) reaches around 1–20% of a conventional ^1^H-NMR [24]. However, recent studies show promising results that indicate Pure Shift methods could be added to the metabolomics toolbox [10,17,28,29,30]. Lopez et al. demonstrated that an untargeted metabolomics approach for *Physalis peruviana* fruits based on SAPPHIRE-PSYCHE revealed good results in the STOCSY and PLS analyses [17]. Furthermore, Santacruz et al. used PSYCHE decoupling for the differentiation of coral extracts, and Bo et al. adapted the method to honey and tea samples [10,28]. All the studies indicated, besides the additional structural information, advantages in the untargeted metabolomics data processing. Those studies handled samples with quantitative differences in metabolites, but just to a certain extent, they also differed in their chemical composition. The suitability of Pure Shift for highly diverse sample sets, for which peak picking and intelligent binning [31] is not possible, has been examined in the study presented here. Until now, it has not been clear to what extent binning has a positive or negative effect on the data processing of PSYCHE spectra. Uniform binning, the data reduction of the spectrum into small integral regions of the same size, often results in signals, especially multiplets of large widths, being unintentionally distributed over several bins, and thus being only represented inadequately by one bin. A better separation of signals could lead to better binning results.

First, the best suitable Pure Shift method was determined, and the parameters were optimized to analyze complex extracts of different *Hypericum* species, which are known to vary in their major secondary metabolites [7]. Second, an optimized bin size was determined for PSYCHE spectra. Finally, the performance of the PSYCHE experiment within multivariate data analysis was addressed in terms of quantifiability, metabolite identification, and applicability.

## 2. Results and Discussion

### 2.1. Comparison of Pure Shift Methods

**Figure 1:**
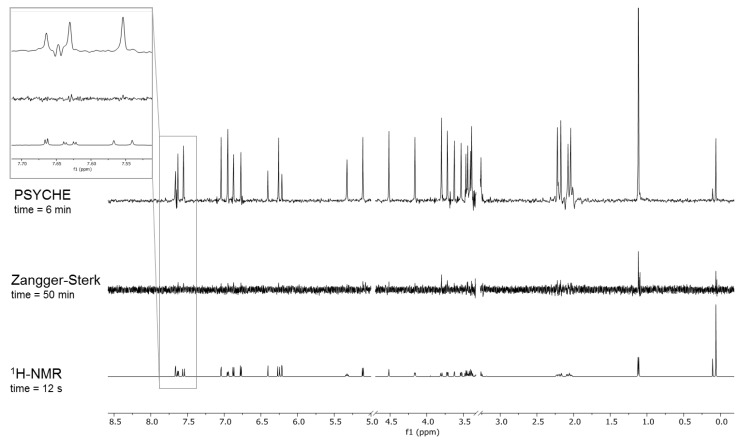
Comparison of the ^1^H-NMR, Zangger–Sterk (PSD1 [19]) and PSYCHE [32] spectra acquired with the default parameter settings (number of scans = 4).

Different Pure Shift methods are known. The most common experiments are the Zangger–Sterk [19] and the PSYCHE methods [32]. Both techniques were tested with a compound mixture of chlorogenic acid (**1**) and rutin (**2**) (for structures and numbering schemes see Figure S.1). The combination of these *Hypericum* constituents was chosen because the coupling constants of the signals cover a wide range, from 2 to 16 Hz [7] (Figure S.1). The first experiment was performed with the spectrometer control software’s (VnmrJ) default settings (pulse sequences given in data depository). Figure 1 shows the Pure Shift spectra in comparison to the conventional ^1^H-NMR. The Zangger–Sterk method (Figure 1) revealed, despite the long measuring time of 50 min, a low signal to noise ratio (SNR), and cannot compete with the sensitivity of the PSYCHE experiment. Additionally, the measuring time of the PSYCHE experiment is nearly ten times shorter than Zangger–Sterk. Therefore, the PSYCHE method was considered for metabolomics experiments, wherein acquisition times should be short, as usually high sample numbers and compound stability are important issues. The homonuclear decoupling was achieved for all signals in the PSYCHE experiment to an acceptable extent. The SNR was lower than in conventional ^1^H-NMR, as already described by Castanar [24]. Furthermore, artifacts (e.g., Figure 1: 7.65 ppm) were generated in the PSYCHE spectrum, so the parameters had to be optimized to obtain a higher spectral quality.

### 2.2. Parameter Optimization of the PSYCHE Experiment

The PSYCHE spectra show decoupled signals and a variety of undesired signals, resulting from strong couplings, “chunking” sidebands, and other artifacts [27]. The experimental parameters were improved to generate spectra with high spectral purity, containing a good ratio between wanted and unwanted signals. Furthermore, the sensitivity, calculated as the quotient of SNR and the measurement time, was considered as a quality parameter. 

**Figure 2:**
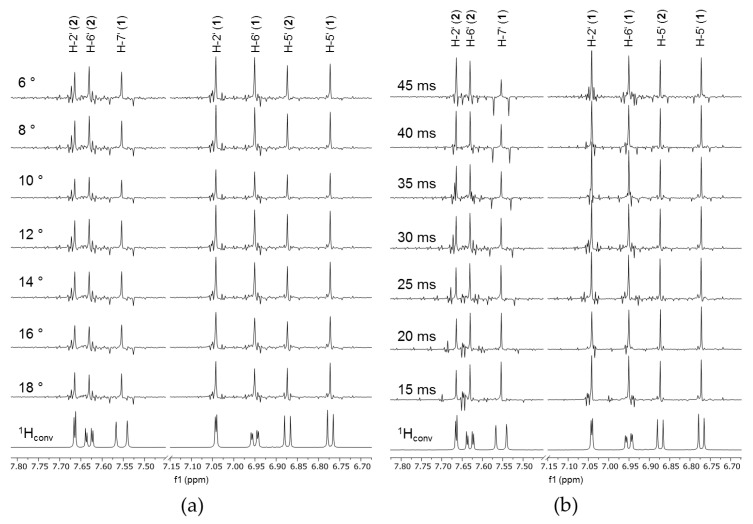
PSYCHE spectra section of compound mixture of chlorogenic acid (**1**) and rutin (**2**) with (**a**) constant τPS (30 ms) and varied pulse angle, and (**b**) constant pulse angle (10°) and varied τPS.

**Figure 3:**
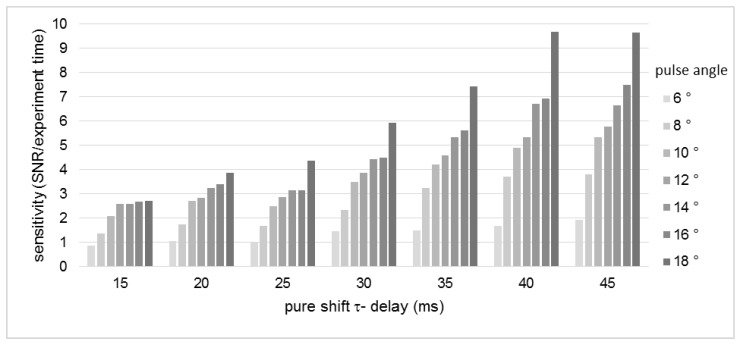
Effect of the variation of pulse angle and τPS on the sensitivity (given as signal to noise ratio (SNR) per experiment time) of the PSYCHE spectra.

The influence of the essential parameters, swept pulse flip angle and Pure Shift tau-delay (τPS = 1/(2sw1)) on spectra quality was tested. The small pulse angle is the fundamental idea that enables the homodecoupling of the PSYCHE experiment [27]. The impact was determined by changing the pulse angle (6°–18°) gradually with a constant τPS (30 ms). The obtained spectra are shown in Figure 2a. The utilization of pulse angles above 10° results in decoupling sidebands, observable at the signal H-5′ of **2** at 6.88 ppm. The calculated sensitivities are summarized in Figure 3. It becomes clear that big pulse angles accompany high SNRs, and thus a better sensitivity is reached. Nevertheless, the use of small pulse angles is a reasonable compromise, since it leads to the acquisition of spectra with a high homodecoupling efficiency [24,27,33]. 

In the next experiments, the τPS was subsequently changed while the pulse angle was kept constant. With the increasing τPS, the experiment time became shorter. While a PSYCHE measurement with a τPS of 5 ms takes 155 min, an experiment with 45 ms takes only 39 min, with the same number of scans of 16. Furthermore, the spectral purity is highly influenced by the τPS, observable in Figure 2b. The suitability of the parameter depends on the coupling constant *J* of the signals. As shown in Figure 2b, the largest *J* (signal H-7′ of **1,** *J* = 15.9 Hz) is successfully decoupled with τPS values up to 20 ms. These findings are in line with the recommendations of Foroozandeh et al., who stated that the sw1 (sw1 = 1/(2τPS)) should be twice the highest *J* in order to decouple [32]. For signals with large *J* values, such as in the case of signal H-7′ (**1**, *J* = 15.9 Hz), it can be clearly seen that the negative sidebands increase with the increasing τPS. These sidebands are caused by chunking, and show up in the distance of sw1 or 1/(2τPS). On the other hand, a small τPS leads to artifacts between signals with strong couplings, such as for compound **2** signals H-2′ (*J* = 2.2 Hz) and H-6′ (*J* = 2.2 Hz, 8.4 Hz). Thus, the choice of a certain τPS value is always a compromise. Further, the COSY type and strong coupling artifacts could be reduced by elongating the duration of the pulse. However, a pulse length of 30 ms, which is consistently reported in the literature [17,28], gave satisfactory results.

In our study, we decided to use a swept pulse flip angle = 10°, a τPS = 15 ms, and a pulse width = 30 ms, in order to enable the decoupling of the larger *J* from the aromatic and olefinic protons of the secondary metabolites. The decision was based on the taxonomic marker compounds described for the *Hypericum* species, such as flavonoids, xanthones, phloroglucinols and organic acids [7,12,15,16]. Furthermore, the influence of homodecoupling on binning was investigated, whereby particularly large coupling constants are problematic due to a higher signal width.

In general, it can be concluded that the spectroscopist determines which signals are better detected and which worse by setting the parameters. This is fine for structure elucidation and targeted approaches, but for untargeted metabolomics, it is definitely a drawback.

### 2.3. Suitability of PSYCHE and ^1^H-NMR for Metabolomics Studies

It was investigated whether PSYCHE has advantages over a conventional ^1^H-NMR during classical metabolomics processing with subsequent multivariate data analysis. In total, 21 *Hypericum* species (Table S.1), represented by 29 genotypes with up to three biological replicates, were analyzed with quantitative ^1^H-NMR, conventional ^1^H-NMR, and a PSYCHE experiment. In contrast to other Pure Shift methods, a PSYCHE spectrum contains quantitative information, and thus can be used for metabolomics experiments [23]. In the metabolomics workflow used, the spectra were baseline corrected and referenced. Then, a uniform binning was applied, which divides the spectrum into bins of the same size over the whole spectral width. Finally, the binned spectra were normalized to the internal standard HMDS and evaluated by principal component analysis (PCA).

#### 2.3.1. Optimization of Bin Size

**Figure 4:**
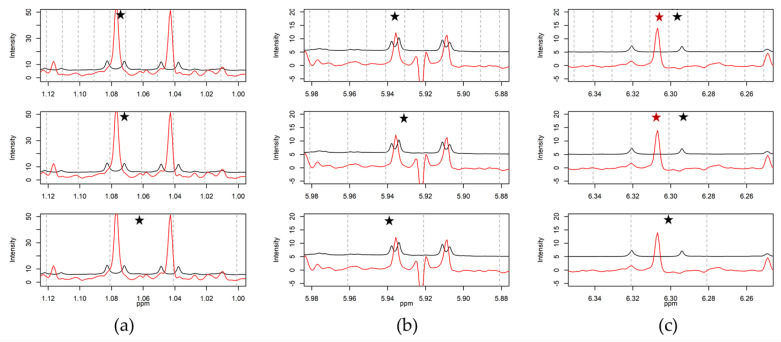
Selected sections of conventional ^1^H-NMR (black) and PSYCHE (red) spectra of a representative *Hypericum perforatum* sample evaluated with different bin sizes to display signals at: (**a**) 1.08 ppm (d, 6.5 Hz) H_3_-12 of hyperforin (**5**); (**b**) 5.94 ppm (d, 2.4 Hz) H-6 of epicatechin/catechin (**4**); and (**c**) 6.31ppm (d, 15.8 Hz) H-8′ of chlorogenic acid (**1**). For each signal, three bin sizes are shown (from up to down: 0.01, 0.02, and 0.04 ppm). The dashed vertical grey line marks the borders of each bin. The chosen bin for evaluation is highlighted with an asterisk.

**Figure 5:**
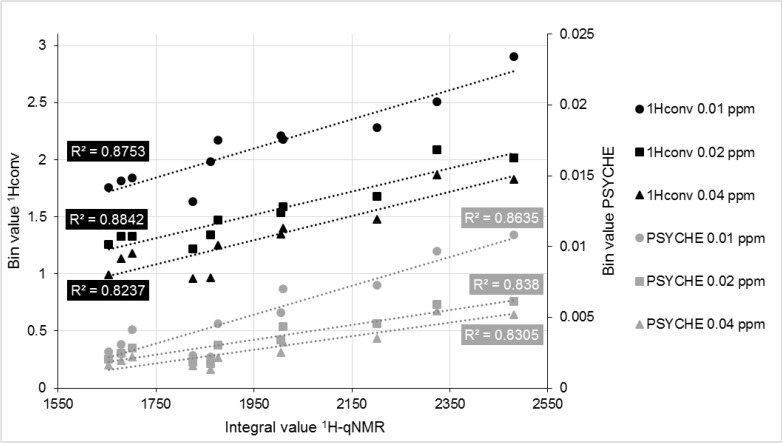
Linear regression between the integral of the signal at 1.08 ppm from the ^1^H-qNMR measurement, and the corresponding bin value of the conventional ^1^H-NMR (1Hconv, black) and the PSYCHE (grey) experiment with different bin sizes. The coefficient of determination, R^2^, was calculated for each graph.

Binning is a form of data reduction. The spectra are cut into sections (bin), and the total integral of each bin is used as the evaluation value. Ideally, a bin completely includes one signal and represents the total integral, and thus the amount of substance in the sample. The integrals measured in the quantitative ^1^H-NMR and the corresponding bin value of the PSYCHE spectrum were compared to estimate the signal representation. The integration of the signals presupposes a baseline separation from other peaks. For the comparison of both methods, eleven samples of five genotypes of *H. perforatum* (including biological replicates) were used. This ensures that no overlap of the signals chosen for quantification occurs, since intraspecifically the quantities of the constituents change, but regularly, not the chemical composition. Nine baseline separated signals derived from seven constituents (Table 1) were integrated and compared to the bins of PSYCHE and conventional ^1^H-NMR (examples shown in Figure 4). Figure 5 visualizes the procedure for the example of the doublet of the methyl group H_3_-12 of hyperforin (**5**) at 1.08 ppm. The integrals and bin values were combined in an XY diagram, and the coefficient of determination for the resulting regression line was used as a quality parameter. This procedure is inspired by Ludwig et al., who evaluated the quantitation of 2D J-resolved NMR experiments [29].

For uniform binning, different bin sizes were used by different authors. We tested the regularly used bin sizes 0.01 ppm [34], 0.02 ppm [35] and 0.04 ppm [8]. In Figure 4 (extended in Figure S.2), it can be seen that the application of the bin size 0.01 ppm often results in split signals. In contrast, bins with boundaries of 0.04 ppm include multiple signals (Figure 4a, 0.04 ppm). Anderson et al. calculated that bins of 0.01 and 0.02 ppm enclose on average one peak per bin, whereas bins of 0.04 ppm contain four [31]. To check if different bin sizes lead to a changing quantitative correlation with the concentration of the selected ingredients, the coefficients of determination (R^2^) were compared (Table S.2). This comparison was performed for conventional ^1^H-NMR and PSYCHE experiments. No significant difference (Wilcoxon–Mann–Whitney, *p* > 0.05) between the bin sizes could be determined (Figure S.3). However, we decided to use 0.02 ppm for further processing, because the R^2^ average was the highest, and the same size was successfully used in other studies [35,36]. 

**Figure 6:**
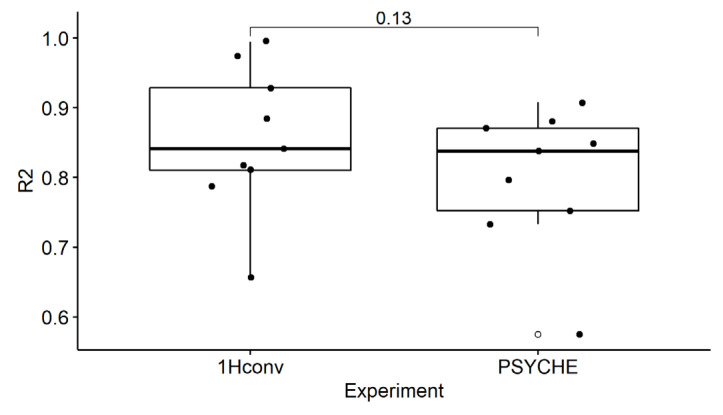
Boxplot of the coefficient of determination (R^2^, Table 1) for the conventional ^1^H-NMR (1Hconv) and the PSYCHE experiment with a bin size of 0.02 ppm. Significance Test: Wilcoxon–Mann–Whitney paired (shown is *p*-value).

One goal of this analysis was to check if PSYCHE may have advantages in comparison to conventional ^1^H-NMR, generated by uniform binning. Figure 6 shows the boxplots of the R^2^ values, generated from the PSYCHE and the conventional ^1^H-NMR experiments. The R^2^ values are not significantly different from each other (Wilcoxon–Mann–Whitney, *p* > 0.05). However, the data from conventional ^1^H-NMR tend to reach slightly higher R^2^ values. This indicates that the PSYCHE experiment has no advantage in terms of quantification during the binning process.

#### 2.3.2. Multivariate Data Analysis of Different Hypericum Species

**Figure 7:**
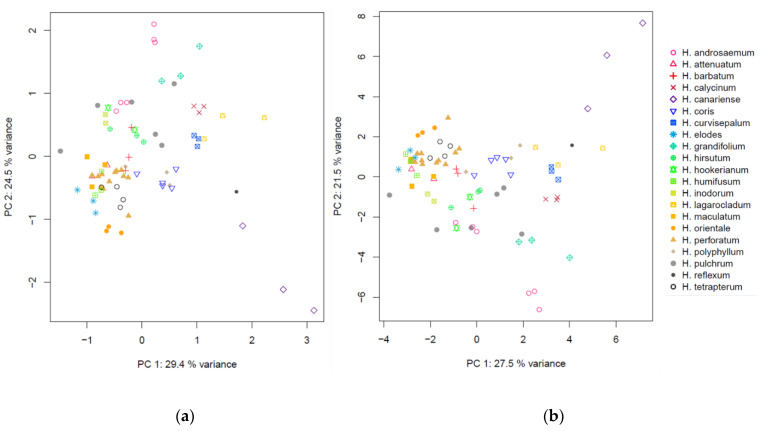
Scores plot of principal component analysis (PCA) of *Hypericum* species based on (**a**) conventional ^1^H-NMR and (**b**) PSYCHE spectra.

For 21 *Hypericum* species with up to three biological replicates, both a conventional ^1^H-NMR and a PSYCHE spectrum were recorded. All data sets were identically processed, as described in the material and methods section. As already known from earlier studies [7,14,16,37,38], the interspecific variance within the genus *Hypericum* is huge. The obtained spectra are highly diverse (Figures S.4 and S.5), so no alignment or peak picking was carried out. The spectra are exceptionally varying in the region of olefinic and aromatic protons (5.5–8.0 ppm), and in signals belonging to phloroglucinol-related resonances (1.0–2.0 ppm). Consequently, the spectra were binned without alignment and peak picking or adaptive binning to avoid forcing unequal signals to fall in the same bin. The scores plot of the PCA performed with conventional ^1^H-NMR data is displayed in Figure 7a. The scores plot based on PSYCHE data demonstrates similar clusters and differs only marginally (Figure 7b). 

The bin tables were freed from bins that did not exceed the limit of detection (LOD, calculated as three times the standard deviation of the noise regions (10–11 ppm)) to exclude bins with no informational content. In the case of PSYCHE data, due to signal narrowing by homodecoupling, the signal width is reduced so that the signal will be expected to be distributed over a lower number of bins; ideally it is found in one bin only. However, the number of bins beyond the LOD was comparable for both methods. Within the bin table of the conventional ^1^H-NMR data, 9%, and in the case of PSYCHE data, 10%, of the bins could be removed. Utilizing the reduced data for PCA, no changes were observed in the first four principal components (PCs), indicating that the separation in PCA is independent of noise bins (Figure S.6). It can be concluded that for the *Hypericum* data set, uniform binning with excluding noise data has, in general, no advantage in comparison to conventional ^1^H-NMR. Santacruz et al. used adaptive intelligent binning, and were able to reduce their initial data matrix of 168 bins from the conventional ^1^H-NMR to 113 bins from the PSYCHE spectra [10]. Although they did not comment on that, this bin reduction could be a reason for the better PLS results based on PSYCHE than with the conventional ^1^H-NMR data. The advantage of less complex spectra is possibly better exploited with adaptive intelligent binning than with uniform binning, and should be considered when comparing samples with mainly quantitative changes. 

**Figure 8:**
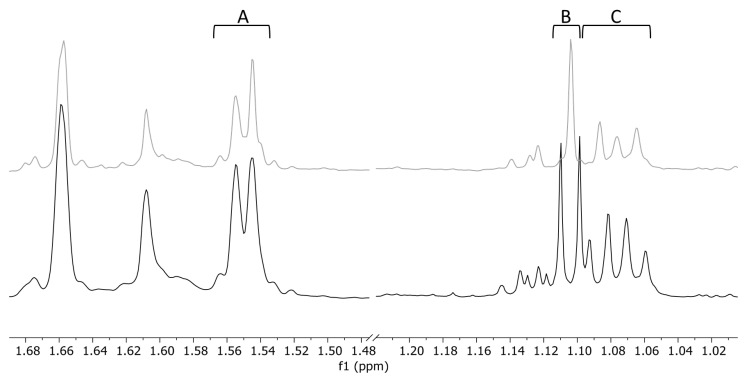
Regions of interest (**A**–**C**) in the conventional ^1^H-NMR spectrum (black) of *Hypericum canariense* where the PSYCHE spectrum (grey) helps to identify signals and their multiplicity.

Even if the noise bin reduction has no impact on the PCA result in the *Hypericum* data set, the PSYCHE spectra simplify the assignment of signals of interest. In Figure 8, three examples of interesting regions are highlighted. Regarding region A (Figure 8), the two peaks at 1.55 ppm could correspond to one doublet with *J* 5.6 Hz, but in the PSYCHE spectrum, it is unambiguous that these are two singlets with similar chemical shifts. In contrast, the signal in region B (Figure 8) is a true doublet (*J* 6.5 Hz), which becomes a singlet by homodecoupling. Intriguing is also region C (Figure 8), where the spectroscopist would initially assume a quartet, but the PSYCHE spectrum reveals that these are three overlapping signals with similar coupling constants. These few examples already show that the PSYCHE experiment can provide important information for the interpretation of overlapping regions.

The application of PSYCHE to complex samples like plant extracts is helpful for structural elucidation, and also reveals when combined with multivariate data analysis, good results. However, the results are comparable to the conventional ^1^H-NMR, and therefore, the PSYCHE will never replace the ^1^H-NMR in this kind of analysis. By adding the PSYCHE experiment to the metabolomics workflow, the total experimental time will elongate, although the PSYCHE acquisition time was herein reduced. However, the time-consuming spectra interpretation and structure elucidationcan be accelerated by including PSYCHE for selected samples, so that the time from data acquisition to answering the research question will be shorter.

In particular, choosing the right parameters for the PSYCHE experiment is difficult, so different types of artifacts are still included. The additional optimization of the gradient amplitude and the duration of the pulse (for a given bandwidth) might also improve the results. Furthermore, new modified pulse sequences can even improve the spectral purity of the basic PSYCHE, like the triple spin-echo PSYCHE [26] and the SAPPHIRE-PSYCHE [17], which shows the great potential of these methods.

## 3. Materials and Methods

### 3.1. Plant Material and Samples

For the metabolomics experiment, 29 genotypes of 21 different *Hypericum* species (Table S.1) were cultivated in the Leibniz Institute of Plant Genetics and Crop Plant Research (IPK) in Gatersleben. The seeds were provided by the genebanks in IPK and Kew Gardens in London. The cultivating processes and conditions are as described in Rizzo et al. [39]. Depending on the germination success, each genotype was represented with up to 6 plants, where one to three biological replicates were generated (Table S.1). In November 2018, the leaves of all the samples were collected into 20 mL grinder polyvials (Zinsser Analytic GmbH, Eschborn, Germany) and immediately frozen in liquid nitrogen. After lyophilization (Martin Christ Gefriertrocknungsanlagen GmbH, Osterode, Germany, 144 h), two stainless steel balls (5 mm) were added to each sample and they were powdered twice for 15 s with the Cryogenic Plant Grinder (Labman, −75 °C, 5% humidity, 30 Hz). A break of 30 s in between avoided the warming of the samples.

### 3.2. Sample Preparation

In total, 40 mg of each plant material was directly extracted with 1 mL methanol-*d*_4_ (99.8%, Deutero GmbH, Kastellaun, Germany), containing 0.935 mM HMDS (hexamethyl disiloxane) as the internal standard. After 30 s of vigorous mixing with vortex genie 2 (Scientific industries, Bohemia, NY, USA), 15 min of ultrasonic bath extraction was applied. The samples were centrifuged for 10 min at 14,000 rpm (5415 C Eppendorf, Eppendorf AG, Hamburg, Germany) to separate the plant powder. Then, 0.73 mL of the supernatants was transferred to Deu-Quant-5-7 NMR tubes (Deutero).

For method development, a mixture of chlorogenic acid (Sigma Aldrich, Steinheim, Germany, 1.73 mg/mL) and rutin (Roth, Karlsruhe, Germany, 3.20 mg/mL) was solved in methanol-*d*_4_.

### 3.3. NMR Data Acquisition

The spectra were recorded on an Agilent VNMRS 600 NMR spectrometer (Varian, Palo Alto, CA, USA) at 25 °C equipped with a 5 mm inverse detection cryoprobe using standard CHEMPACK 8.1 pulse sequences s2pul, PS1D and PSYCHE (for parameter and pulse sequences see data repository, respectively) implemented in the Varian VNMRJ 4.2A software. The signals were referenced to internal HMDS at 0.062 ppm. The compound mixture and plant extract spectra were measured with a spectral width of 10 and 13 ppm, respectively. Quantitative ^1^H-NMR (^1^Hq) spectra were measured with the following parameters: pulse angle = 90°, relaxation delay (d1) + acquisition time (at) = 30.0 s, number of scans (nt) = 128, and digital resolution = 0.37 Hz/point. Conventional ^1^H-NMR (^1^H) spectra were aquired with the following: pulse angle = 30°, d1 + at = 3.0 s, nt = 40, and digital resolution = 0.95 Hz/point. PS1D (Zangger–Sterk) spectrum were recorded with d1 + at = 3 s, nt = 4, digital resolution = 0.37 Hz/point, B1max = 0.1526 kHz, pulse width = 17 ms, and gradient = 1.5 G/cm. The PSYCHE spectra were acquired with d1 + at = 1.7 s, nt = 4, and digital resolution = 1.47 Hz/point for parameter adjustment, and d1 + at = 3.0 s, nt = 16, and digital resolution = 0.97 Hz/point for metabolomics measurements. The applied double saltire Chirp pulses were used with swept pulse flip angle = 10°, τPS = 15 ms, pulse width = 30 ms, and gradient = 1.0 G/cm.

### 3.4. NMR Data Processing and Data Analysis

The data processing was carried out with MestreNova (12.0.4-220023, Mestrelab Research, S.L., Santiago de Compostela, Spain). After automatic Fourier transformation with the standard VNMRJ window function and zero filling, phase correction and baseline correction (Bernstein polynomial fit) were applied. The signal to noise ratios (SNR) of *H. perforatum* extracts were calculated with MestreNova, as the ratio of the intensity of the signal δ 6.70–6.78 ppm to the standard deviation of the noise (δ 8.1–10.0 ppm). For the metabolomics experiments, the spectra were reduced to integrated regions of equal width (0.01 ppm, 0.02 ppm, or 0.04 ppm). A binned data table (.csv) was generated. 

Data analysis was performed on the binned data of the conventional ^1^H-NMR and PSYCHE spectra with R version 3.5.1 [40]. The bins corresponding to spectral regions of residual methanol (δ 3.27–3.33 ppm) and water (δ 4.7–5.0 ppm) were removed. Bin values were normalized to the quantitative internal standard HMDS. For principal component analysis (PCA), the pcaMethods (1.78.0) package was utilized. For the reduced data set, all bins below the limit of detection (LOD) in all samples were removed. The LOD was determined as three times the standard deviation of the noise (δ 10 to 11.0 ppm and −1.0 to −0.2 ppm).

Raw data of NMR measurements, used pulse sequences, as well as the processed bin tables are freely available in the RADAR repository [41].

## 4. Conclusions

In this study, the PSYCHE experiment was optimized for the *Hypericum* plant extracts, representing complex mixtures of various compound classes. The swept pulse angle and the pure shift τ-delay were adapted to get satisfactory results for coupling constants larger than 6 Hz. This required a measuring time of 32 min, which is 16 times longer than the conventional ^1^H-NMR. However, the method presented here was much shorter than the ones reported before. The extraction of quantitative information was possible from the binned PSYCHE spectra, and was not significantly different to the results from the binned conventional ^1^H-NMR. With the uniform binning method, which is used for the spectra of compositionally different *Hypericum* extracts, the gain in resolution through homodecoupling did not affect multivariate data analyses, such as PCA. So, the PCA of both methods leads to similar results. However, the PSYCHE spectra are able to support the data interpretation and compound identification of the NMR spectra by simplifying the crowded spectral parts. This is the real power of the Pure Shift methods. Therefore, we recommend the implementation of Pure Shift methods in the follow-up experiments of the NMR metabolomics workflow, and the use of them to interpret the data. 

## Figures and Tables

**Table 1:** Summary of the coefficient of determination (R^2^) after linear regression between ^1^H-qNMR integrals and bin values of different experiments with bin size 0.02 ppm (Supplement S.2). Numbering scheme of compounds is shown in Figure S.1.

Compound	Assignment	δ (ppm) Multiplicity (*J*)	R^2^ of Experiment	
			^1^Hconv	PSYCHE
Chlorogenic acid (**1**)	H-8′	6.31 d (15.8 Hz)	0.6553	0.8485
Chlorogenic acid (**1**)	H-2′	7.05 d (2.1 Hz)	0.7865	0.5748
Rutin (**2**)	H-6′′′	1.12 d (6.2 Hz)	0.8176	0.7331
Hyperoside (**3**)	H-2′	7.83 (d 2.2 Hz)	0.9288	0.8810
Epicatechin/Catechin (**4**)	H-6	5.94 d (2.4 Hz)	0.9740	0.7974
Epicatechin/Catechin (**4**)	H-2′	6.97 d (1.9 Hz)	0.8104	0.7525
Hyperforin (**5**)	H_3_-12	1.08 d (6.5 Hz)	0.8842	0.8380
Sucrose (**6**)	H-3′	4.09 d (8.2 Hz)	0.9945	0.9080
Shikimic acid (**7**)	H-4	4.36 m (*ν*_1/2_ 4.7 Hz)	0.8412	0.8706

## Supplementary Materials

The following are available online. Figure S.1: Chemical structures and corresponding ^1^H-NMR data. Table S.1: List of plant material. Figure S.2: Graphical representation of different bin sizes for selected quantified signals. Table S.2: Quantitative correlation of bins of different signals. Figure S.3: Boxplot of correlation coefficient for different experiments with different bin sizes. Figure S.4: Stacked conventional ^1^H-NMR spectra of *Hypericum* species. Figure S.5: Stacked PSYCHE spectra of *Hypericum* species. Figure S.6: PCA scores plots of different experiments.
