# Supplementary material for: PSYCHE—A Valuable Experiment in Plant NMR-Metabolomics
Source: Molecules. 2020 Nov 4;25(21):5125. doi: 10.3390/molecules25215125 (PMC7662903; doi:10.3390/molecules25215125)
Supplement: Supplementary file 1 [file molecules-25-05125-s001.pdf]

Supplement: PSYCHE - a valuable experiment in plant NMR-metabolomics

Pauline Stark [1,*], Caroline Zab [1], Andrea Porzel [1], Katrin Franke [1], Paride Rizzo [2] and Ludger A. Wessjohann[1,*]

[1] Department of Bioorganic Chemistry, Leibniz Institute of Plant Biochemistry, Weinberg 3, 06120 Halle/Saale, Germany

[2] Department of Bioorganic Chemistry, Leibniz Institute of Plant Genetics and Crop Plant Research, Corrensstraße 3, 06466 Seeland, Germany

* Correspondence: Ludger.Wessjohann@ipb-halle.de; Tel.: + 49 (0) 345 5582 1300
Pauline.Stark@ipb-halle.de

Content

Figure S.1: Chemical structures and corresponding ^{1}H-NMR data 2

Table S.1: List of plant material 3

Figure S.2: Graphical representation of different bin sizes for selected quantified signals 4

Table S.2: Quantitative correlation of bins of different signals 5

Figure S.3: Boxplots of correlation coefficients for different experiments with varied bin sizes 6

Figure S.4: Stacked conventional ^{1}H-NMR spectra of *Hypericum* species 7

Figure S.5: Stacked PSYCHE spectra of *Hypericum* species 8

Figure S.6: PCA scores plots of selected experiments 9

Figure S.1: Chemical structures and corresponding ^{1}H-NMR data

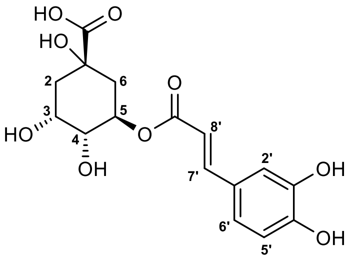

Chlorogenic acid (**1**)

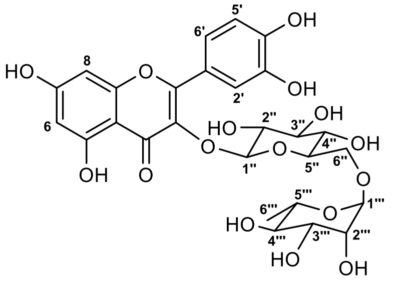

Rutin (**2**)

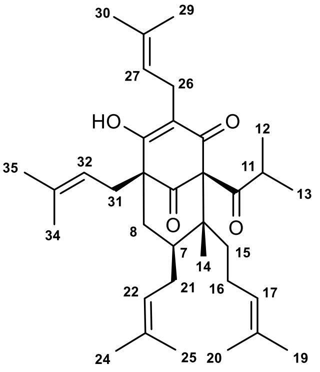

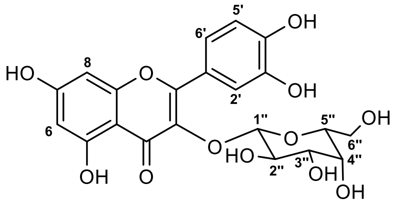

Hyperoside (**3**)

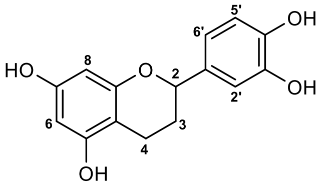

Catechin/Epicatechin (**4**)

Hyperforin (**5**)

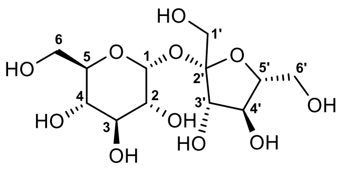

Sucrose (**6**)

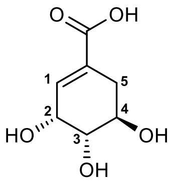

Shikimic acid (**7**)

Chlorogenic aicd (1): ^{1}H NMR (600 MHz, Methanol-*d*$_4$) δ 7.55 (d, *J* = 15.9 Hz, 1H, H-7‘), 7.04 (d, *J* = 2.1 Hz, 1H, H-2‘), 6.95 (dd, *J* = 8.2, 2.1 Hz, 1H, H-6‘), 6.77 (d, *J* = 8.2 Hz, 1H, H-5‘), 6.26 (d, *J* = 15.9 Hz, 1H, H-8‘), 5.33 (m, 1H, H-3), 4.16 (m, 1H, H-5), 3.72 (m, 1H, H-4), 2.19 (m, 2H, H-6), 2.05 (m, 2H, H-2).
Rutin (2): ^{1}H NMR (600 MHz, Methanol-*d*$_4$) δ 7.66 (d, *J* = 2.2 Hz, 1H, H-2‘), 7.63 (dd, *J* = 8.4, 2.2 Hz, 1H, H-6‘), 6.87 (d, *J* = 8.4 Hz, 1H, H-5‘), 6.40 (d, *J* = 2.1 Hz, 1H, H-8), 6.21 (d, *J* = 2.1 Hz, 1H, H-6), 5.11 (d, *J* = 7.7 Hz, 2H, H-1‘‘), 4.52 (d, *J* = 1.6 Hz, 1H, H-1‘‘‘), 3.83 – 3.21 (H-2‘‘-H-6‘‘, H-2‘‘‘-H-5‘‘‘), 1.12 (d, *J* = 6.2 Hz, 3H, H-6‘‘‘).
Hyperoside (3): ^{1}H NMR (600 MHz, Methanol-*d*$_4$) δ 7.84 (d, *J* = 2.2 Hz, 1H, H-6‘), 7.58 (dd, 1H, H-2‘), 6.87 (d, 1H, H-5‘), 6.41 (d, *J* = 2.2 Hz, 1H, H-8), 6.21 (d, *J* = 2.2 Hz, 1H, H-6), 5.17 (dd, *J* = 7.2, 2.5 Hz, 2H, H-1‘‘), 3.93 – 3.37 (m, 5H, H-2‘‘-H-6‘‘).
Epicatechin (4): ^{1}H NMR (600 MHz, Methanol-*d*$_4$) δ 6.97 (d, *J* = 1.9 Hz, 1H, H-2‘), 6.79 (dd, *J* = 8.2, 2.0 Hz, 1H, H-5‘), 6.75 (d, *J* = 8.2 Hz, 1H, H-6‘), 5.93 (d, *J* = 2.4 Hz, 1H, H-6), 5.91 (d, *J* = 2.3 Hz, 1H, H-8), 4.58 (s, 1H, H-2), 4.17 (m, 1H, H-3), 2.86 (dd, *J* = 16.7, 4.6 Hz, 1H, H-4a), 2.73 (dd, *J* = 16.7, 3.0 Hz, 1H, H-4b).
Hyperforin (5): ^{1}H NMR (600 MHz, Methanol-*d*$_4$) δ 5.21 (t, 1H, H-27), 5.10 (t, 2H, H-17), 5.06 (t, 1H, H-22), 5.01 (t, 1H, H-32), 3.18 (m, 1H, H-26a), 3.03 (m, 1H, H-26b), 2.42 (dd, *J* = 14.6, 7.8 Hz, 1H, H-31a), 2.32 (dd, *J* = 14.3, 7.7 Hz, 1H, H-31b), 1.70 – 1.54 (m, 24H, H-19, H-20, H-24, H-25, H-29, H-30,H-34, H-35), 1.45 – 1.16 (m, 10H, H-7, H-8, H-11, H-15, H-16, H-21), 1.05 (d, *J* = 6.6 Hz, 3H, H-12), 1.07 (d, *J* = 6.6 Hz, 3H, H-13), 0.93 (s, 3H, H-14).
Sucrose (6): ^{1}H NMR (600 MHz, Methanol-*d*$_4$) δ 5.38 (d, *J* = 3.8 Hz, 1H, H-4a), 4.09 (m, 1H, H-2‘), 3.63 (d, *J* = 12.3 Hz, 1H, H-8), 3.59 (d, *J* = 12.3 Hz, 1H, H-6), 3.41 (dd, *J* = 9.8, 3.8 Hz, 2H, H-4b).
Shikimic acid (7): ^{1}H NMR (600 MHz, Methanol-*d*$_4$) δ 6.79 (m, 1H, H-2), 4.37 (m, 1H, H-3), 3.98 (m, 1H, H-4), 3.67 (m, 1H, H-5), 2.69 (m, 1H, H-6a), 2.19 (m, 1H, H-6b).

Table S.1: List of plant material

Specification of *Hypericum* plant material used and naming scheme

Genotype	Species	Section	biological replicates
IPK_2	*H. attenuatum Fisch. ex* Choisy	*Hypericum*	3
IPK_3	*H. grandifolium* Choisy	*Androsaemum*	3
IPK_4	*H. calycinum* L.	*Ascyreia*	3
IPK_5	*H. inodorum* Mill.	*Androsaemum*	3
IPK_10	*H. polyphyllum* Boiss. & Balansa	*Olympia*	3
IPK_24	*H. perforatum* L.	*Hypericum*	1
IPK_27	*H. androsaemum* L.	*Androsaemum*	3
IPK_28	*H. maculatum* Crantz	*Hypericum*	3
IPK_33	*H. coris* L.	*Coridium*	3
IPK_34	*H. hirsutum* L.	*Taeniocarpium*	3
IPK_35	*H. orientale* L.	*Crossophyllum*	3
KEW_02	*H. hookerianum* L.	*Ascyreia*	2
KEW_04	*H. canariense* L.	*Webbia*	3
KEW_05	*H. reflexum* L.f.	*Adenosepalum*	1
KEW_09	*H. perforatum* L.	*Hypericum*	3
KEW_10	*H. perforatum* L.	*Hypericum*	2
KEW_12	*H. perforatum* L.	*Hypericum*	3
KEW_15	*H. tetrapterum* Fr.	*Hypericum*	2
KEW_19	*H. humifusum* L.	*Oligostema*	3
KEW_22	*H. elodes* L.	*Elodes*	3
KEW_23	*H. barbatum* Jacq.	*Drosocarpium*	3
KEW_26	*H. perforatum* L.	*Hypericum*	3
KEW_27	*H. pulchrum* L.	*Taeniocarpium*	3
KEW_28	*H. pulchrum* L.	*Taeniocarpium*	3
KEW_33	*H. curvisepalum* N. Robson	*Ascyreia*	3
KEW_37	*H. lagarocladum* N. Robson	*Ascyreia*	3
KEW_44	*H. androsaemum* L.	*Androsaemum*	3
KEW_45	*H. tetrapterum* Fr.	*Hypericum*	2
KEW_46	*H. coris* L.	*Coridium*	2

Figure S.2: Graphical representation of different bin sizes for selected quantified signals

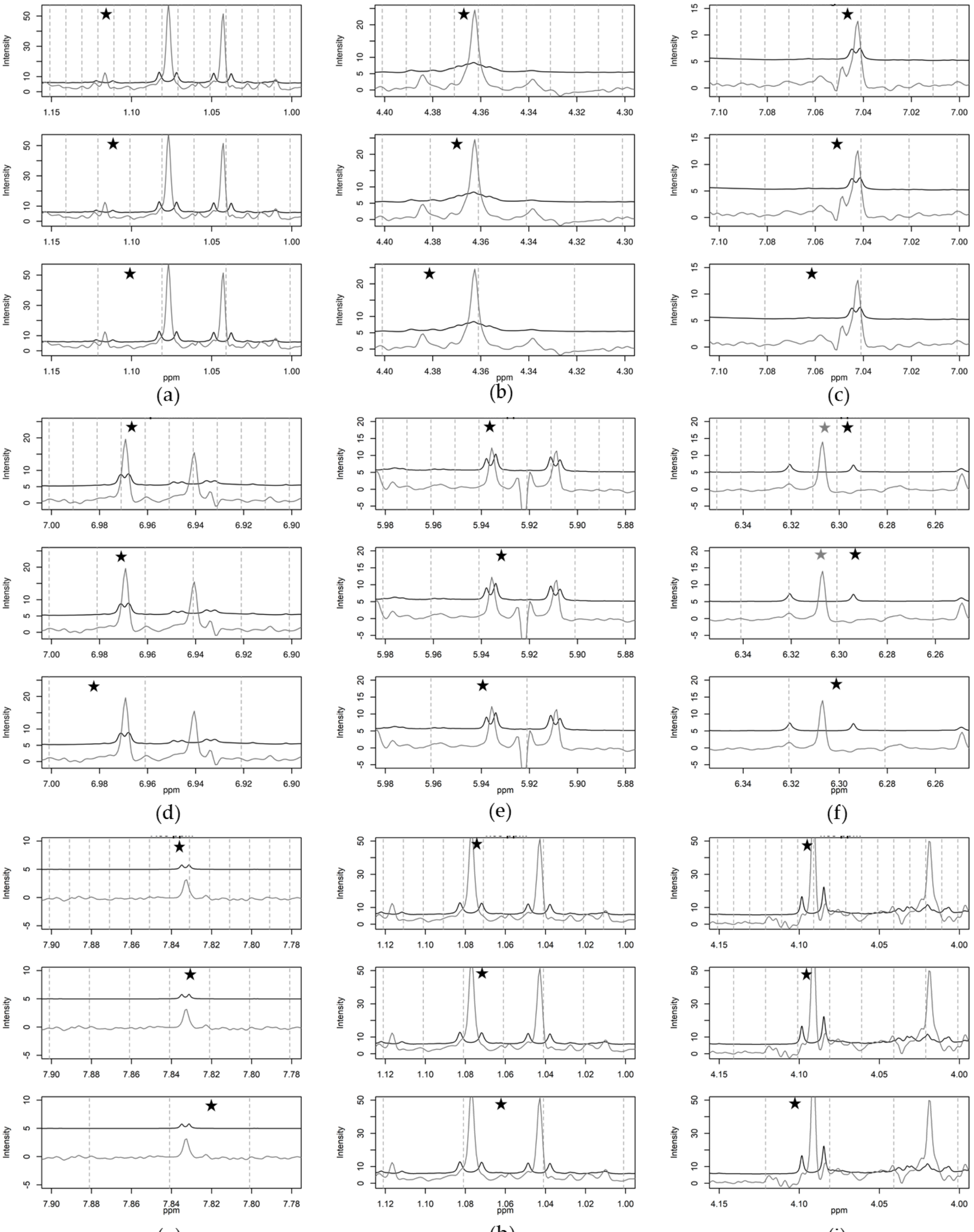

Figure 1. Selected sections of conventional ^{1}H-NMR (black) and PSYCHE (grey) spectra of a representative *H. perforatum* sample to display signal at: (**a**) 1.12 ppm (d, 6.2 Hz) corresponding to rutin (**2**, H-6’’’); (**b**) 4.36 ppm (m) corresponding to shikimic acid (**7**, H-4); (**c**) 7.05 ppm (d, 2.1 Hz) corresponding to chlorogenic acid (H-2’ (**d**) 6.97 ppm (d, 1.9 Hz) corresponding to epicatechin/catechin (H-2’); (**e**) 5.94 ppm (d, 2.4 Hz) corresponding to epicatechin/catechin (H-6); (**f**) 6.31ppm (d, 15.8 Hz) corresponding to chlorogenic acid (H-8'); (**g**) 7.83 ppm (d, 2.2 Hz) corresponding to hyperoside (H-2’); (**h**) 1.08 ppm (d, 6.5 Hz) corresponding to hyperforin (H_3-12); (**i**) 4.09 ppm (d, 8.2 Hz) corresponding to sucrose (H-3’). For each signal, three bin sizes are shown (from up to down: 0.01, 0.02, and 0.04 ppm). The dashed vertical grey lines mark the borders of each bin. The bin selected for the evaluation is marked with an asterisk.

Table S.2: Quantitative correlation of bins of different signals

Summary of the coefficient of determination (R^2) of linear regression between integral (^1H qNMR) and bin values of experiments with different bin sizes.

Compound	Assignment	δ [ppm] multiplicity (J [Hz])	Bin size [ppm]	R^2 of experiment	
				$^1H_{conv}$	PSYCHE
Chlorogenic acid (**1**)	H-8′	6.31 d (15.8 Hz)	0.01	0.7822	0.7200
			0.02	0.6553	0.8485
			0.04	0.8667	0.801
Chlorogenic acid (**1**)	H-2′	7.05 d (2.1 Hz)	0.01	0.7537	0.5365
			0.02	0.7865	0.5748
			0.04	0.744	0.5861
Rutin (**2**)	H-6′′′	1.12 d (6.2 Hz)	0.01	0.8166	0.6229
			0.02	0.8176	0.7331
			0.04	0.8311	0.3024
Hyperoside (**3**)	H-2′	7.83 d (2.2 Hz)	0.01	0.902	0.8884
			0.02	0.9288	0.881
			0.04	0.9178	0.8518
Epicatechin/Catechin (**4**)	H-6	5.94 d (2.4 Hz)	0.01	0.9925	0.9156
			0.02	0.9740	0.7974
			0.04	0.9824	0.8204
Epicatechin/Catechin (**4**)	H-2′	6.97 d (1.9 Hz)	0.01	0.7791	0.7526
			0.02	0.8104	0.7525
			0.04	0.7896	0.7627
Hyperforin (**5**)	H_3-12	1.08 d (6.5 Hz)	0.01	0.8753	0.8635
			0.02	0.8842	0.8380
			0.04	0.8237	0.8305
Sucrose (**6**)	H-3′	4.09 d (8.2 Hz)	0.01	0.9294	0.5399
			0.02	0.9945	0.9080
			0.04	0.8928	0.8542
Shikimic acid (**7**)	H-4	4.36 m ($\nu_{1/2}$ 4.7 Hz)	0.01	0.7929	0.8025
			0.02	0.8412	0.8706
			0.04	0.8115	0.8762

Figure S.3: Boxplots of correlation coefficients for different experiments with varied bin sizes

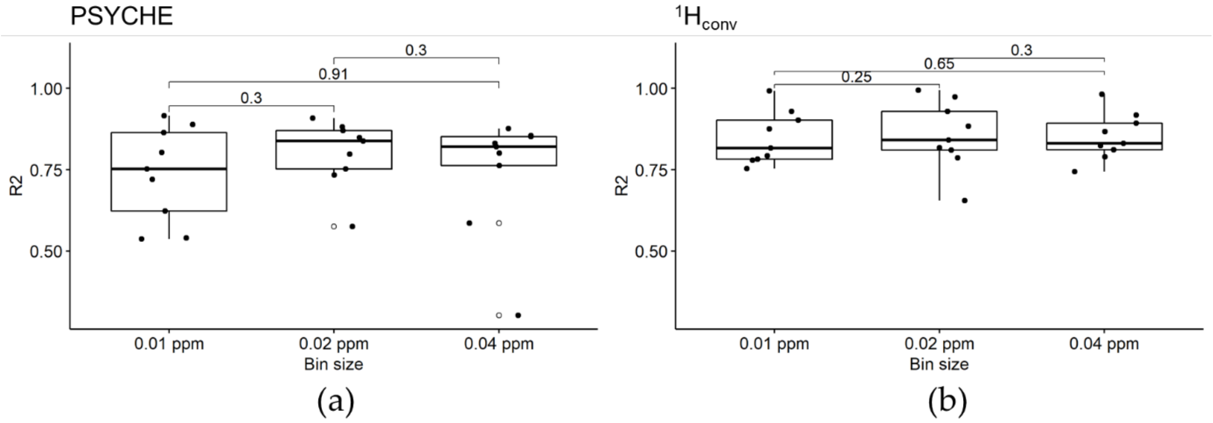

Boxplot of R^2 with varied bin sizes of (a) PSYCHE and (b) conventional ^{1}H-NMR (^{1}Hconv).
Significance Test: Wilcoxon-Mann-Whitney paired (shown is the p-value).

Figure S.4: Stacked conventional ^{1}H-NMR spectra of *Hypericum* species

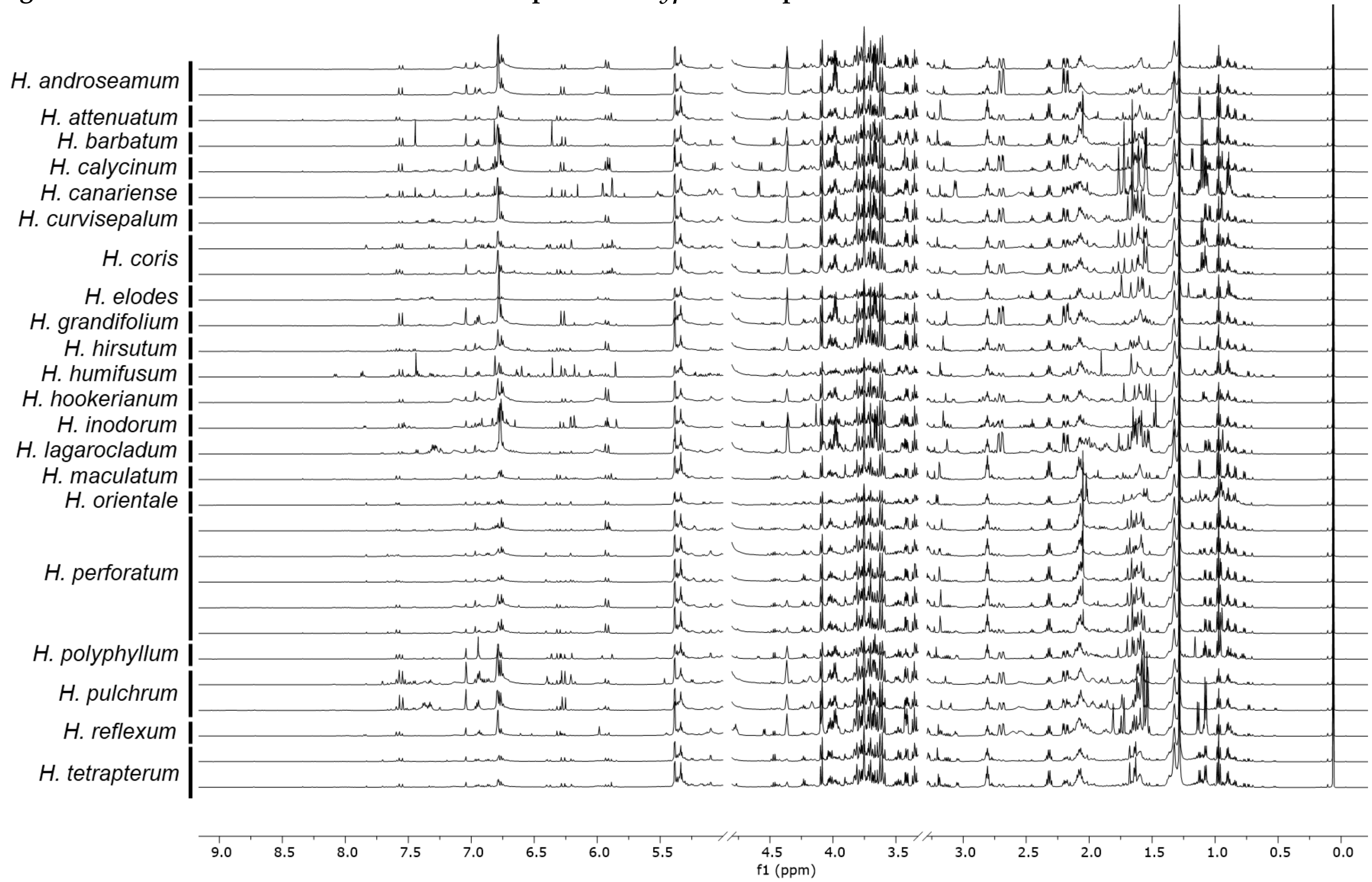

Stacked conventional ^{1}H-NMR spectra of one biological replicate for each genotype.

Figure S.5: Stacked PSYCHE spectra of *Hypericum* species

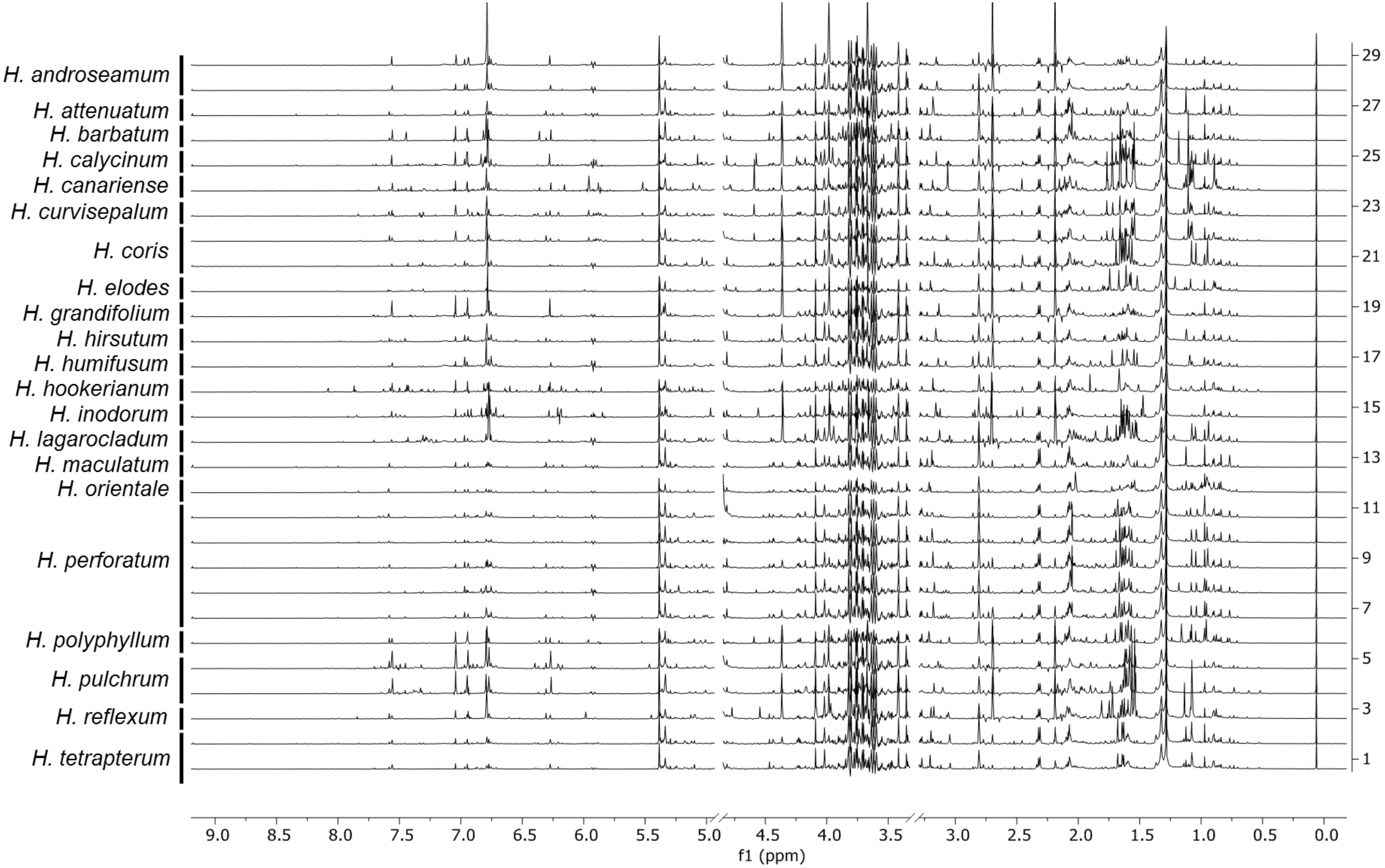

Stacked PSYCHE spectra of one biological replicate for each genotype.

Figure S.6:   PCA scores plots of selected experiments

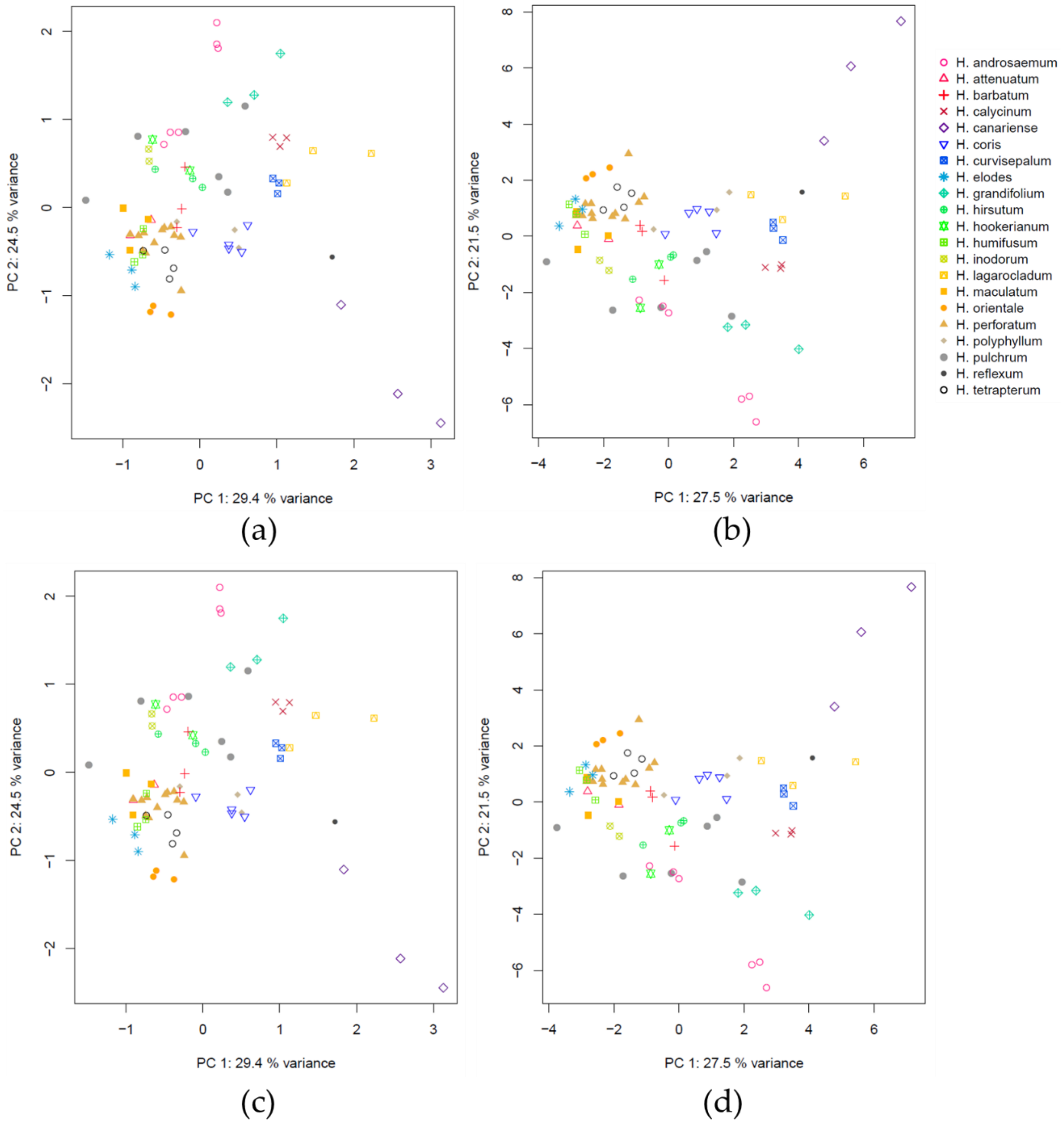

Scores plots of principal component analyses (PCAs) of *Hypericum* species based on (**a**) conventional ^{1}H-NMR and (**b**) PSYCHE spectra. Scores plots of PCAs, based only on bins with signals higher than the limit of detection (three times standard deviation of noise) of (**c**) conventional ^{1}H-NMR and (**d**) PSYCHE spectra.
